# Spirochæta Pallida of Schaudiun

**Published:** 1906-04-07

**Authors:** 


### SPIROCHÆTA PALLIDA OF SCHAUDINN.

The discovery of a spirillum in syphilitic patients was announced by Schaudinn and Hoffmann, of Berlin, in May 1905. They had found the organism they claimed not only in the surface papules and chancres, but also in the deeper tissues of the lymphatic glands. The announcement was of interest in that bacteriological advancement is extending largely in the direction of organisms higher in the scale than bacteria and cocci. The plasmodium of malaria and the trypanosome of sleeping sickness are among those which are recognised generally as the causal agents of the diseases mentioned. While within recent times attempts have been made to show that a plasmodium is the cause of cancer, and Schaudinn himself considers that a protozoon may prove the organism at fault in yellow fever.

There have been announcements before of bacteria in syphilis, which, however, have not stood the required tests. Niessen described such a bacillus. Wallsch, in investigating cases of syphilis for this bacillus, has found it in chancres and inguinal glands, and secondary manifestations of the disease, while in nonsyphilitics it was not to be found. It gave inconclusive results on inoculation, and Wallsch concluded that in itself it was not the actual causal agent of syphilis.

With this disappointing result before us, any new discovery of an organism has to be accepted with caution, and the majority of writers upon the new spirochæte, while admitting its presence in syphilitic patients, are yet loath to draw absolute conclusions of its specificity.

Metchnikoff and Roux, however, conclude from their experiments that they are justified in the belief that syphilis is a chronic spirillosis due to the spirochæte described by Schaudinn. This standpoint is perhaps more tenable from the fact that Metchnikoff and others have been able to remove the infected virus from the fluid used by filtration, and have destroyed its activity by raising it to a temperature of 60° C. But other observers have considered that while it is impossible that the spirillum should be grown upon artificial media, and hence all of Koch's postulates with regard to specificity be carried out, it is premature to do more than agree to its presence in syphilitics and to leave the possibility of its being other than one member of a symbiotic condition, or an inert parasite causing no symptoms, for the future to decide.

It is of interest in this connection to note that Castellani has found a very similar spirillum in yaws, which disease has raised so much discussion as to its identity with or distinction from syphilis. The investigations of these organisms will be watched with the greatest interest, but the fear may be expressed that the spirochæta pallida of Schaudinn may be found to play the same harmless rôle which is exhibited by the spirochæta refringens, a similar organism, which, according to Heidingsfeld and Markley, is to be found constantly in the stagnating lesions of conditions other than syphilitic.

